# Modeling the Impact and Costs of Semiannual Mass Drug Administration for Accelerated Elimination of Lymphatic Filariasis

**DOI:** 10.1371/journal.pntd.0001984

**Published:** 2013-01-03

**Authors:** Wilma A. Stolk, Quirine A. ten Bosch, Sake J. de Vlas, Peter U. Fischer, Gary J. Weil, Ann S. Goldman

**Affiliations:** 1 Department of Public Health, Erasmus MC, University Medical Center Rotterdam, Rotterdam, The Netherlands; 2 Infectious Diseases Division, Department of Internal Medicine, Washington University School of Medicine, St. Louis, Missouri, United States of America; 3 Department of Epidemiology and Biostatistics, The George Washington University School of Public Health and Health Services, Washington, D. C., United States of America; Imperial College London, United Kingdom

## Abstract

The Global Program to Eliminate Lymphatic Filariasis (LF) has a target date of 2020. This program is progressing well in many countries. However, progress has been slow in some countries, and others have not yet started their mass drug administration (MDA) programs. Acceleration is needed. We studied how increasing MDA frequency from once to twice per year would affect program duration and costs by using computer simulation modeling and cost projections. We used the LYMFASIM simulation model to estimate how many annual or semiannual MDA rounds would be required to eliminate LF for Indian and West African scenarios with varied pre-control endemicity and coverage levels. Results were used to estimate total program costs assuming a target population of 100,000 eligibles, a 3% discount rate, and not counting the costs of donated drugs. A sensitivity analysis was done to investigate the robustness of these results with varied assumptions for key parameters. Model predictions suggested that semiannual MDA will require the same number of MDA rounds to achieve LF elimination as annual MDA in most scenarios. Thus semiannual MDA programs should achieve this goal in half of the time required for annual programs. Due to efficiency gains, total program costs for semiannual MDA programs are projected to be lower than those for annual MDA programs in most scenarios. A sensitivity analysis showed that this conclusion is robust. Semiannual MDA is likely to shorten the time and lower the cost required for LF elimination in countries where it can be implemented. This strategy may improve prospects for global elimination of LF by the target year 2020.

## Author Summary

The Global Program to Eliminate Lymphatic Filariasis (LF) employs annual mass drug administration (MDA) of antifilarial drugs to reduce infection rates in populations and interrupt transmission. While this program is working well in many countries, progress has been slow in others, and some countries have not yet started MDA programs. We used computer simulation modeling and cost projections to study how increasing MDA frequency from once to twice per year would affect program duration and costs. Our results suggest that semiannual MDA is likely to reduce the time required to eliminate LF by 50% and reduce total program costs (excluding the cost of donated drugs) in most situations. For these and other reasons, we expect semiannual MDA to be superior to annual MDA in most endemic settings. Semiannual MDA should be considered as a means of accelerating LF elimination in areas where it can be implemented, because this may improve prospects for global elimination of LF by the target year 2020.

## Introduction

The Global Program to Eliminate Lymphatic Filariasis (GPELF) was launched in 2000 with the aim of eliminating lymphatic filariasis (LF) as a public health problem by 2020 [1]. The recommended strategy is to treat entire at-risk populations annually with a single dose of ivermectin and albendazole (IVM+ALB) in sub-Sahara Africa or with diethylcarbamazine and albendazole (DEC+ALB) in other regions for a minimum of 5 years [2]. Mapping studies suggest that mass drug administration (MDA) is needed in 72 endemic countries [3].

As indicated in the GPELF 2010 progress report, progress toward LF elimination varies widely between countries [3]. Some countries started their MDA programs early and may have already interrupted LF transmission, while other countries lag behind [3]. Nineteen countries had not yet started MDA, and geographical coverage was incomplete in 24 others. Reasons cited for slow progress in some areas included major logistic challenges, political instability, conflict, and co-endemicity with *Loa loa* [4]. Also, results from ongoing MDA programs have sometimes been disappointing. Sentinel site data collected after 5 years of annual MDA show that microfilaria (mf) prevalence had dropped to 0% in about two-thirds of sentinel sites sampled. However, mf rates had decreased by less than 50% in 10% of the sites sampled [4].

With the goal of LF elimination by 2020 in mind, it is now important and timely to study whether elimination programs can be accelerated. A straightforward option would be to increase the frequency of MDA from once per year (annual) to twice per year (semiannual). While increasing MDA frequency might be expected to shorten the time required for elimination, the magnitude of this effect is uncertain. Only one study directly compared the impact of annual and semiannual MDA and this was for brugian filariasis: semiannual MDA with DEC alone caused a more rapid decline in mf prevalence than annual treatment. However, the duration of this study was too short to support conclusions regarding elimination [5]. Results from other studies of semiannual MDA are difficult to interpret, because they did not provide results from a control area with annual MDA [6], [7], [8].

For decision-making, it is also important to consider how costs per year and overall costs for LF elimination programs are likely to change if MDA frequency is increased. Of course, costs per year will increase, but they will not necessarily double, and the cumulative cost for the entire program may even decline. The costs of twice yearly MDA have not been formally studied for LF or other neglected tropical diseases. However, they can be projected from detailed cost data by activity and cost item that are available for yearly MDA for LF and soil-transmitted helminthiasis [9], [10], [11], [12].

We have used the well-established simulation model LYMFASIM to estimate the number of treatment rounds and duration of MDA programs that would be needed to eliminate LF with annual and semiannual MDA in different settings. Simulations were performed for typical endemic areas in West Africa (with IVM+ALB treatment and *Anopheles* transmission) and in India (with DEC+ALB treatment and *Culex* transmission) with different pre-control endemicity levels and MDA coverage rates. In addition, we have compared projected costs of annual or semiannual MDA, both per year and for the total required duration of LF elimination programs.

## Methods

### Estimating the required duration of annual and semiannual MDA programs

#### The LYMFASIM simulation model

The LYMFASIM model describes the transmission of *Wuchereria bancrofti* parasites in a dynamic human population, to predict time trends in infection indicators and the effects of control programs. The LYMFASIM model employs the technique of stochastic microsimulation [13], to allow inclusion of chance processes and variation in important human characteristics and behaviors. The mathematical background of the model was described in detail by Plaisier et al. [14]. Here we provide a brief description.

The model simulates a closed population, consisting of a discrete number of individuals. The population composition changes over time due to birth and death of individuals. The history of infection and disease is simulated at the level of the individual human, taking account of individual variation in exposure to mosquito bites (age-related or random), life span, immune responsiveness to infection, compliance with MDA programs, and responsiveness to treatment. Worms in humans are also simulated individually, allowances made for separate sexes and variable life spans. Mature female worms produce mf during their reproductive lifespan at certain rates when there are male worms present in the human body. Because of all these factors, worm loads and mf intensity vary between human individuals, as well as their contribution to the average force of infection working on the population. In calculating the latter, the model considers the vector species-specific non-linear relationship between mf intensity in human blood and the average number of mf engorged by biting mosquitoes.

The primary outcomes of the model are predicted trends in the mf prevalence rate and mean mf intensity in the population. These outcomes are based on mf counts for all individuals in the population, while taking account of test characteristics that determine sampling variation and the possibility of false-negative test results. This makes simulation outcomes directly comparable to field data. For the present study, we assumed that mf counts were done by microscopic examination of a 20-µl or 60-µl thick smear of night finger-prick blood.

#### Modeling specific endemic regions

We used previously developed LYMFASIM model variants for India and West Africa. The India model describes the epidemiology of bancroftian filariasis in India which is transmitted by *Culex quinquefasciatus*, and it was tested against longitudinal data from Pondicherry, India [15]. The West Africa model describes the epidemiology in African regions where *Anopheles-*species are the only vectors of LF. This model was validated against cross-sectional data [16]. We refer to the original publications for details about model parameter values and the procedures used for parameter estimation and validation.

Table 1 lists values for key parameters for the India and West Africa model variants. Many parameters were assumed to have the same value in both models, such as the parameters describing the parasite life cycle, age-variation in exposure, and variability in mf counts. Assumptions about immunity differ between the two model variants, to explain the different epidemiological patterns. In Pondicherry, India, mf prevalence and mf intensity declined in the oldest age groups, and a form of acquired immunity was incorporated into the model. Here, we assume that immunity, triggered by incoming L3 larvae, reduces the probability for incoming L3 larvae to develop successfully into adult worms. Since such a decline in mf prevalence is unusual in Africa and mf prevalence rates in this region tend to be higher than those in India [17], the West Africa model does not include acquired immunity.

**Table 1:** Values of LYMFASIM parameters in models for India and West Africa.

	Parameter value	
Description	India	West Africa
Average number of mosquito bites/adult person/month, for areas with low, intermediate and high pre-control Mf prevalence	1600,1950,2700	430, 485, 650
Exposure at birth, fraction of maximum exposure^a^	0.26	0
Age at which exposure reaches maximum^a^	19.1 years	20.0 years
Shape parameter for γ distribution describing individual variation in exposure (mean = 1; a higher value indicates less variability)	1.13	0.26
Function that specifies the number of L3-larvae developing in mosquitoes after a single blood meal as a function of human mf density in 20 µl of blood (*m*)	(0.089 *m*)/(1+6.6 *m*)	1.67(1-exp(-(0.027 *m*)^1.51^)
Success ratio: the fraction of incoming L3 larvae that survive and develop into mature adult worms.	1.03×10^−3^	8.8×10^−3^
Fraction of L3 larvae, from 1 blood meal, released by a mosquito when it bites	0.1	0.1
Mean life span of parasites in human host	10.2 years	10.0 years
Shape parameter for the Weibull distribution that describes variation in parasite life span	2.0	2.0
Duration of immature stage of parasite in human host	8 months	8 months
Fraction of microfilariae surviving per month	0.9	0.9
Number of Mf produced/female parasite/month/20 µl of peripheral blood	0.61	0.58
Scale parameter for sigmoid function relating strength of anti-L3 immunity to experience of infection by L3	5.89×10^−5^	n.a.
Shape parameter for γ distribution describing individual variation in ability to develop anti-L3 immunity	1.07	n.a.
Duration of immunological memory for anti-L3 immunity	9.6 months	n.a.
Clumping factor for the negative binomial distribution describing variation in mf-counts in 20 µL blood smears from an individual with given mf density. Between brackets: idem, for 60 µL blood smears	0.345 (1.035)	0.33 (0.99)

The table lists parameters related to transmission and parasite development, for which the values may vary between the models. See original publications for a full justification of the parameter values [15], [16].

n.a. = not applicable.

a Exposure increases with age until a maximum is achieved at a certain age; exposure remains at its maximum level thereafter.

The relationship between human blood mf density and mosquito parasite uptake also differs between the two models, reflecting known differences between the vector species. The India model for *Culex quinquefasciatus* assumes ‘limitation’ in this relationship: “L3 yield” in mosquitoes (i.e. the number of L3 developing in mosquitoes per mf in the human blood) declines monotonically with increasing mf density in the human blood and saturation occurs at high mf densities. The West Africa model assumes ‘facilitation’: the L3 yield initially increases with mf density in human blood, although limitation still causes saturation at higher mf densities [16].

The West Africa model assumes relatively strong inter-individual variation in exposure to mosquito bites (indicated by the low value for the shape parameter of the gamma distribution in the West Africa model), to capture the variation between people in mf density in the human blood. The India model assumes less variability in exposure, because the variability in human mf density is partly attributed to acquired immunity and the associated individual differences in immune responsiveness. Further, the monthly biting rate (mbr, defined as average number of mosquito bites per adult person per month) is known to vary between communities, and we considered different values as explained below.

#### Simulated scenarios and MDA assumptions

We performed simulation experiments to estimate the duration of MDA required to achieve LF elimination using different values for key parameters in the Indian and West African models. Mbr values were chosen to simulate communities with low, intermediate or high pre-control mf prevalence levels that are encountered in these regions. The simulated pre-treatment mf prevalence levels (based on 60 µL blood smears) were 7.7%, 11.5% and 15% for India and 12.5%, 20% and 27.5% for West Africa. Corresponding values for 20 µL blood smears would be approximately 5%, 7.5% and 10% for India and 9%, 14% and 20% for West Africa.

For each setting, we simulated a range of treatment scenarios that varied with respect to simulated treatment regimens (DEC+ALB or IVM+ALB), frequency of treatment (annual or 6-monthly), treatment coverage (55%, 70% or 85% of the total population; constant over time), and the number of treatment rounds (1, 2, …, 20 rounds). Compliance with offered treatment was simulated as a partially systematic process. That is to say, it is neither completely random (where each person has the same chance to get treated in each round) nor completely systematic (where all individuals either take all or none of the treatments), but somewhere in between. The simulated proportion of systematic non-compliers (i.e. those who never take treatment) for a given number of treatment rounds is not fixed; it depends on overall treatment coverage levels; the proportion of systematic non-compliers in the total population increases when the overall coverage declines, and vice versa. This mechanism fairly represented the attendance pattern of a mass treatment program for onchocerciasis in Asubende, Ghana [18].

Baseline treatment efficacy assumptions were based on expert opinion and ultrasound studies [19], [20], [21], as justified elsewhere [22]. A single treatment with DEC+ALB was assumed to kill 70% of mf and to kill or permanently sterilize 65% of adult worms. Similarly, a single treatment with IVM+ALB was assumed to kill 100% of mf and to kill or permanently sterilize 35% of adult worms. These treatment effects were assumed to occur just after treatment. Further, we assumed that there is no inter-individual variation in treatment effects and that the treatment efficacy is the same in all treatment rounds.

#### Estimating required program durations

To calculate the probability of LF elimination for a certain setting and treatment scenario, we performed repeated simulations (n = 1000), all with the exact same assumptions. We recorded for each run whether elimination was reached (defined as mf prevalence <0.1%, measured 60 years after the first MDA to allow for slow extinction of the parasite population when mf prevalence was brought below its breakpoint level). The elimination probability was defined as the percentage of runs that reached this outcome. The required number of MDA rounds for elimination was estimated as the lowest number of MDA rounds that resulted in a ≥99% probability of elimination. For annual MDA programs, the estimated required number of MDA rounds equals the duration of MDA in years. For semiannual MDA programs, the duration of MDA in years equals the number of MDA rounds divided by 2.

### Estimating the costs of annual and semiannual MDA

We estimated the costs of MDA for LF programs with annual and semiannual treatment from the perspective of the endemic country government. The cost analysis covers financial and economic costs. The financial costs are the costs of all inputs purchased in cash for MDA, including purchased MDA drugs, materials and supplies, ministry of health personnel salaries, and per diem payments for community drug distributors [11]. Economic costs also include the costs of donated drugs for MDA (India: albendazole; Burkina Faso: ivermectin, albendazole). Costs were calculated for a target population of 100,000 eligible persons in three steps.

#### Step 1. Estimate the cost per treatment round for annual MDA from published data

For India, we based our calculations on published data on the total cost per treatment round as estimated by Ramaiah and Das [23]. The total costs as published were measured in 1996 and included the cost of purchased DEC (50 mg tablets). The India program relied on government health workers for drug distribution and did not use volunteers. This study did not include central government costs for planning. It was carried out at the district level and it measured personnel time at the district level.

For West Africa we used recently published data from Burkina Faso on total costs of MDA (measured in 2002) excluding the cost of donated drugs [11]. We ignored cost data from the first round of MDA in Burkina Faso, because of possible bias due to extra start-up costs. The value of personnel time devoted to MDA in Burkina Faso was based on data collected from Ministry of Health personnel in the national LF program plus regional and district health personnel who participated in the program. [9]. Data from Ghana, from the same publication, were not considered, because they were based on a lower number of data points.

We used a series of calculation steps to estimate the relative cost in 2009 for treating a population with 100,000 eligible persons in both India and West Africa, while correcting for salary or per diem changes, inflation since the original cost study, and potential programmatic changes (see Table 2 for details).

**Table 2:** Calculation steps for estimating the cost of a single mass drug administration (MDA) round.

	India	Burkina Faso	Reference
1. Total cost of MDA as reported, including the cost of drugs (US$, base year^a^ value)	70,412		[23]
2. Total cost of MDA as reported, excluding costs of drugs (US$, base year value)		110,000	[11]
3. Population at risk	2,269,477	2,613,000	[11], [23]
4. Percentage of the population at risk that is eligible for treatment (%)	90	85	
5. Cost per 100,000 eligibles, incl. the cost of drugs (US$, base year value)	3,447^b^	n.a.	
6. Cost per 100,000 eligibles, excl. the cost of drugs (US$, base year value)	808^c^	4,953	
7. As 6), (US$, comparative value in 2009)	1,139^d^	9,299^d^	
8. As 7) after correction for recent programmatic and salary changes, excl. cost of drugs	2,710^e^	12,378^f^	
9. as 8), incl. the cost of any purchased drugs	3,634^g^	n.a.	
10. as 9), incl. the cost of purchased and donated drugs	5,834^h^	434,578^i^	

The table displays the source data and describes all steps that were taken to estimate the cost of a single MDA round per 100,000 eligibles.

n.a. = not applicable.

a The term base year refers to the year in which cost were originally measured (1996 for India, 2002 for West Africa).

b Calculated from 1), 3) and 4), assuming that drugs (50 mg DEC tablets) were purchased for all eligible persons.

c For India: cost of DEC (50-mg tablets; 5.2 tablets p.p. on average; 0.026 US$ p.p. on average) were subtracted.

d Correction for inflation, using the annual deflators as published by the World Bank [24], i.e. the rate of price change in the economy as a whole. The amount under 6) was first converted back to local currency using the base year conversion rate. Then we applied the correction for inflation between the base year and 2009. The new amount was reconverted into US dollars using the 2009 conversion rate. Average annual inflation in India was about 5% between 1996 and 2009. The average annual inflation between 2002 and 2009 in Burkina Faso was 9%.

e We assume that sensitization efforts in India are intensified to achieve higher coverage, as studied elsewhere [25], [26]. Associated extra costs (for personnel and supplies) would be 0.009 US $ per person in 2002, or 0.015 US$ per eligible if adjusted to 2009 values.

f Volunteer remuneration has changed. In 2002, volunteers were paid for 2 days of training only, not distribution. By 2010 Burkina volunteers were remunerated for about 2.5 days training and 7 days distribution; the daily rate remained the same. [sources: [11] and personal communications from program directors in Burkina Faso in 2011].

g In India, DEC has to be purchased by the government, at 0.00924 US% p.p. on average (for 100 mg tablets, 2.75 tablets p.p. on average).

h Donated drug: albendazole (0.022 US$ p.p.).

i Donated drugs: albendazole (0.022 US$ p.p.) and ivermectin (4.2 US$ p.p. on average).

#### Step 2. Estimate the average cost per year and treatment round for semiannual treatment

Firstly, the cost of treating a population with 100,000 eligibles once (the result of step 1 above) was split up by program activity (sensitization, drug distribution, etc.) and cost item (personnel, supplies, transportation, equipment and facilities). Information about this for India was available from Krishnamoorthy et al [9]. For West Africa, we used the information presented by Goldman et al [11], supplemented by more detailed tables than included in the previous publication. The relative cost of some activities and cost items were increased, in line with step 9 of Table 2. Also, in calculating the 2009 drug costs, we used current prices for DEC purchased by the Indian government and current donor valuations for ivermectin and albendazole. The list of activities and cost items for India and West Africa was slightly different, because of differences in program organization and study design choices made by the authors.

Secondly, we made assumptions on the relative increase in cost per year by activity, if MDA were to be provided twice instead of once per year. We assumed that no extra efforts and costs would be required for planning/administration, training, and surveillance/laboratory activities. Costs for enumeration of populations at risk, drug distribution, and for supervision and adverse reaction monitoring would double, with the exception that no extra capital expenses (for equipment and facilities) would be incurred. Sensitization (social mobilization) would also be repeated, and we assumed that the associated cost of supplies would double (items such as posters, radio and newspaper advertisements), but that costs for personnel and transportation involved in social mobilization would increase by only 35%; again no extra costs for capital expenses are assumed. Our assumptions, regarding which activities are repeated in semiannual MDA programs, were based in part on costing studies performed for soil-transmitted helminth control programs [27], [28]. Additional information came from the observed choices of program managers in Burkina Faso, during a 2010 budgeting exercise for twice annual distributions for onchocerciasis in highly endemic districts (A. Goldman, unpublished observations). The average cost per round for semiannual MDA (overall, or by activity and cost item) was then calculated as one half of the cost per year.

#### Step 3. Estimate the cumulative cost of MDA programs with annual or semiannual MDA, considering the total required program duration and discounting

Overall program and economic costs were calculated, taking account of the total required program duration as predicted by LYMFASIM. The overall costs were discounted at a rate of 3% to adjust for a preference to delay cost to the future (some further explanation about discounting can be found in the supporting information text S1). Separate calculations were made including and excluding the cost of donated drugs. We assumed that drugs would be purchased or provided for all people eligible for MDA and that any unused drugs are wasted: i.e. they are not used in later MDA rounds, because they were either distributed and not consumed or they were lost, damaged, or expired [9], [26], [29].

### Sensitivity analysis

We studied the extent to which key assumptions affect conclusions regarding the relative cost of the two MDA schedules (once or twice yearly MDA) in a univariate sensitivity analysis. On the cost side, we assessed the effect of changing the discount rate to 0% or 6% instead of 3%, the effect of including the cost of donated drugs, and we considered the scenario where drugs are only bought for people who are actually treated instead of for all eligibles (with the idea that any remaining drugs would be stored and used in a later round). These factors do not influence the number of rounds required, but they may affect the total costs of annual and semiannual treatment programs and influence policy decisions.

With respect to the simulations, we examined the impact of changing assumptions regarding the efficacy of drugs on adult worms. This may affect the total number of treatment rounds (and total costs) required for LF elimination programs with annual or semiannual MDA. The fraction of worms assumed to be killed or permanently sterilized after each treatment was varied with low, medium (baseline) and high values (50%, 65%, and 80% for DEC+ALB, and 20%, 35% and 50% for IVM+ALB). Further, we studied the impact of including variability in this parameter, so that the fraction of worms killed or sterilized varies randomly between individuals in each treatment cycle and within individuals in different treatment cycles. The variation is described by a beta distribution with the mean equal to the baseline fraction of worms killed/sterilized and standard deviation equal to 0.3.

## Results

### Illustrative examples of simulation results

**Figure 1:**
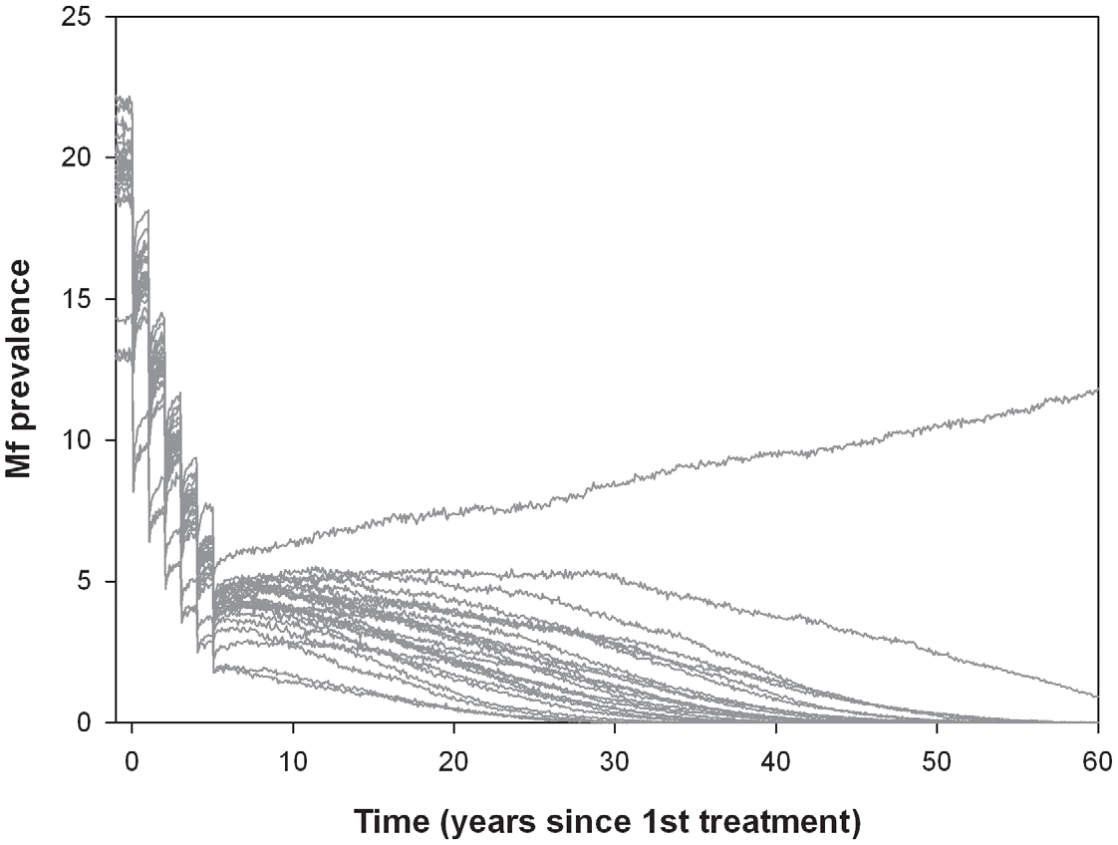
Simulated trends in mf prevalence (%) after mass drug administration. The presented trends are for an African setting with pre-control mf prevalence around 20%, where 6 rounds of annual mass drug administration with IVM+ALB were provided starting at time 0. Coverage was 70% and drug efficacy was quantified according to our baseline assumptions. The figure displays the trend of 25 runs, simulated by LYMFASIM, all with the same input assumptions. Variation in the outcomes is due to stochasticity.

Figure 1 shows an example of model-predicted trends in mf prevalence. The presented trends are for a West African area with a pre-control mf prevalence of 20%. Six rounds of annual MDA with IVM+ALB were provided starting at time 0. Coverage was 70% and drug efficacy was quantified according to our baseline assumptions. The figure displays the trend of 25 runs that were all conducted with the same input assumptions. Variation in the outcomes is due to stochasticity. In this example, 1 out of 25 runs showed recrudescence after stopping MDA; one other run seemed to be moving to elimination, but this was not yet achieved. The probability of elimination in this case was 23/25 (92%).

**Figure 2:**
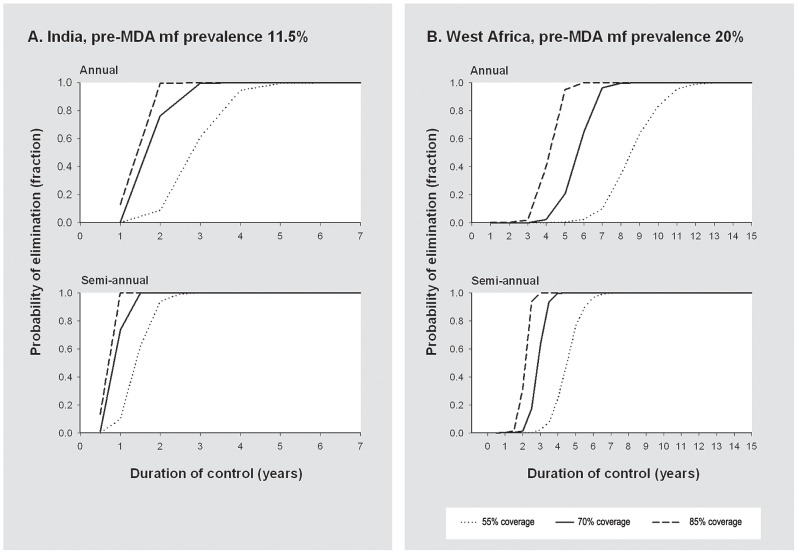
Probability of elimination in relation to the duration of mass drug administration. Panel A shows the results for an Indian setting with a pre-control mf prevalence of about 11.5%, for annual and semiannual mass drug administration and for different coverage levels (percentage of the total population that is treated per round). Similarly, panel B shows the results for an African setting with a pre-control mf prevalence of about 20%. The indicated mf prevalence levels are for diagnosis with 60 µL night blood smears.

Figure 2 illustrates how the model-predicted probability of LF elimination increases with the number of MDA rounds provided. Results are shown for the India and West Africa model variants, for annual and semiannual MDA, and for different coverage levels.

### Estimated cost per treatment round

Table 3 shows the expected costs per treatment round by activity and cost item for annual and semiannual MDA in both regions. Providing semiannual instead of annual MDA reduces the cost per MDA round. This cost reduction is 11% for India and 18% for West Africa, if costs of donated drugs are excluded from the analysis. The cost reduction is smaller if donated drug costs are included (7% for India and only 1% for West Africa).

**Table 3:** Costs per round for annual and semiannual mass drug administration, by activity and cost item.

		India			West Africa		
		Annual	Semiannual		Annual	Semiannual	
		Cost per round ( = cost per year)	Cost per year	Average cost per round	Cost per round ( = cost per year)	Cost per year	Average cost per round
Planning^a^	Personnel	43	43	22	1,903	1,903	952
	Supplies	0	0	0	41	41	21
	Transportation	8	8	4	360	360	180
	Equipment/facilities	9	9	5	237	237	118
	***Total***	***60***	***60***	***30***	***2,541***	***2,541***	***1,271***
Training	Personnel				991	991	495
	Supplies				107	107	53
	Transportation				143	143	71
	Equipment/facilities				2	2	1
	***Total***				***1,242***	***1,242***	***621***
Sensitization^b^	Personnel	684	923	462	262	354	177
	Supplies	823	1,646	823	52	103	52
	Transportation	318	430	215	64	86	43
	Equipment/facilities	18	18	9	103	103	52
	***Total***	***1,843***	***3,017***	***1,508***	***481***	***647***	***323***
Enumeration	Personnel	227	453	227			
	Supplies	60	121	60			
	Transportation	0	0	0			
	Equipment/facilities	0	0	0			
	***Total***	***287***	***574***	***287***			
Drug distribution^c^^,^ d	Personnel	318	636	318	4,770	9,540	4,770
	Supplies (excl. drug)	0	0	0	2,777	5,554	2,777
	DEC (purchased)	924	1,848	924	-	-	-
	ALB (donated)	2,200	4,400	2,200	2,200	4,400	2,200
	IVM (donated)	0	0	0	420,000	840,000	420,000
	Transportation	49	98	49	71	142	71
	Equipment/facilities	57	57	29	82	82	41
	***Total***	***3,549***	***7,040***	***3,520***	***429,900***	***859,718***	***429,859***
Supervision	Personnel	40	79	40			
	Supplies	0	0	0			
	Transportation	26	51	26			
	Equipment/facilities	29	29	15			
	***Total***	***94***	***160***	***80***			
Surveillance/laboratory	Personnel				305	305	153
	Supplies				1	1	1
	Transportation				34	34	17
	Equipment/facilities				0	0	0
	***Total***				***340***	***340***	***170***
Adverse reaction monitoring	Personnel				73	147	73
	Supplies				0	1	0
	Transportation				0	0	0
	Equipment/facilities				0	0	0
	***Total***				***74***	***147***	***74***
**Total cost (incl. all drugs)**		***5,834***	***10,851***	***5,425***	**434,578**	**864,636**	**432,318**
**Excl the donated drugs (ALB for India, IVM+ALB for Afr)**		***3,634***	***6,451***	***3,225***	**12,378**	**20,236**	**10,118**

Costs per year and per treatment round for annual and semiannual mass drug administration programs, per 100,000 eligible persons, in 2009 US$. Cost for West Africa were based on detailed data from Burkina Faso. See Table 2 for data sources and calculation steps.

Abbrevations: DEC = diethylcarbamazine, ALB = albendazole, IVM = ivermectin.

a including administration for West Africa.

b including training of personnel for India.

c including supervision and enumeration for West Africa.

d It is assumed that drugs were purchased for all persons eligible for MDA.

### Total program cost taking account of predicted duration of MDA

Table 4 shows the number of treatment rounds for achieving a 99% probability of elimination, under our baseline assumptions. This number is highly dependent on treatment coverage and pre-treatment mf prevalence rates, but it does not depend much on the frequency of treatment (annual or semiannual). In most circumstances, therefore, the total duration of semiannual MDA is about half of that for annual MDA. In very unfavorable circumstances (areas with high baseline infection rates and very low MDA coverage), one extra MDA round may be required to reach elimination with semiannual MDA.

**Table 4:** Number of treatment rounds required for elimination and total costs of mass drug administration programs.

			# rounds required		Program costs (USD ×1000)	
Setting	Pre-treatment mf prevalence	Coverage (%)	Annual	Semiannual	Annual	Semiannual
India	7.7%	55	3	3	10.6	9.6
		70	2	2	7.2	6.5
		85	2	2	7.2	6.5
	11.5%	55	5	5	17.1	15.8
		70	3	3	10.6	9.6
		85	2	2	7.2	6.5
	15%	55	9	10	29.1	30.4
		70	5	5	17.1	15.8
		85	3	3	10.6	9.6
West Africa	12.5%	55	7	7	79.4	68.2
		70	5	5	58.4	49.4
		85	4	4	47.4	39.9
	20%	55	11	12	118.0	112.9
		70	7	7	79.4	68.2
		85	6	6	69.1	59.0
	27.5%	55	>20^a^	>20^a^	n.a.	n.a.
		70	13	14	135.6	129.9
		85	9	9	99.3	86.5

Results are shown for Indian and West African scenarios, for varying pre-control mf prevalence levels (based on diagnosis with 60 µL blood smears), for annual and semiannual mass drug administration with varying coverage levels. Coverage is defined as the percentage treated out of the total population (including non-eligibles). Total program costs are estimated for a total population of 100,000 eligible persons, based on the estimated total cost per treatment round as presented in Table 3. The costs of donated drugs are excluded (albendazole for India, ivermectin and albendazole for West Africa), but costs of any drugs that have to be purchased by the government are included (DEC for India). The discount rate for future costs was assumed to be 3%. Costs are in 2009 US$ ×1000.

Abbrevations: n.a. = not available.

a Situation unfavorable for elimination.

The total costs of MDA programs depend on the cost per round, the required number of MDA rounds, and thereby also on local circumstances and coverage rates. Table 4 shows estimated total costs for LF elimination programs, assuming an annual discount rate of 3% for future costs. In this analysis, which does not count the cost of any donated drugs, projected costs of semiannual MDA are almost always lower than costs of annual MDA. In West Africa, this is even true when semiannual MDA requires one more MDA round, because of the large reduction in cost per round. In the single India scenario where semiannual MDA required one more round than annual MDA, the projected total program costs were comparable for annual and semiannual MDA.

### Sensitivity analysis

Table 5 and Table 6 summarize the results of the sensitivity analyses for India and West Africa. The tables show the ratio of total program costs for semiannual MDA over annual MDA under varied assumptions. This ratio shows which approach is less expensive (with values <1 indicating that semiannual MDA is cheaper and vice versa), and it provides an indication of the relative cost difference. Changing the discount rate (0% or 6%) had little impact on the projected total costs of semiannual and annual MDA programs and their ratios. Its effect increased with the duration of MDA, and a higher discount rate tends to favor the slower annual MDA programs. But the total program cost of semiannual MDA was lower or comparable to the cost of annual MDA in all scenarios.

**Table 5:** Sensitivity analysis: impact of cost assumptions on the relative cost of semiannual/annual mass drug administration (MDA).

			Ratio of total program costs, with between brackets the estimated total program costs for semiannual over annual MDA (in US$ * 1000)					
Region			India			West Africa		
Pre-control mf prevalence			7.7%	11.5%	15%	12.5%	20%	27.5%
No of MDA rounds required for elimination, semiannual/annual			2/2	3/3	5/5	5/5	7/7	14/13
Assumptions in cost calculations								
Discount rate (fraction)	Donated drugs cost	Purchasing drugs						
0.03^a^	excl^a^	all eligibles^a^	0.90 (6.5/7.2)	0.91 (9.6/10.6)	0.92 (15.8/17.1)	0.85 (49/58)	0.86 (68/79)	0.96 (130/136)
0	excl	all eligibles	0.89 (6.5/7.3)	0.89 (9.7/10.9)	0.88 (16.1/18.2)	0.82 (51/62)	0.82 (71/87)	0.88 (142/161)
0.06	excl	all eligibles	0.92 (6.5/7.1)	0.92 (9.5/10.3)	0.95 (15.4/16.2)	0.87 (48/55)	0.90 (66/73)	1.03 (120/116)
0.03	incl	all eligibles	0.95 (10.9/11.5)	0.95 (16.1/17.0)	0.96 (26.5/27.5)	1.03 (2112/2050)	1.05 (2915/2789)	1.17 (5549/4760)
0.03	excl	treated individuals only	0.88 (6.0/6.8)	0.90 (9.0/10.0)	0.91 (14.8/16.2)	n.r.	n.r.	n.r.
0.03	incl	treated individuals only	0.93 (9.5/10.1)	0.94 (14.1/15.0)	0.95 (23.1/24.2)	1.03 (1748/1698)	1.04 (2412/2311)	1.16 (4592/3944)

The values in the table are the ratio of total program costs, for semiannual MDA/annual MDA. This ratio shows which approach is less expensive (with values <1 indicating that semiannual MDA is less expensive and vice versa), and it provides an indication of the relative differences in cost. Between brackets, the total program costs estimates are given for semiannual/annual MDA programs, in 2009 US$ ×1000. Results are shown for Indian and West African settings, with varying pre-control mf prevalence levels. Coverage of MDA was assumed to be 70% per round (percentage of total population). The number of treatment rounds required for elimination differs between these settings (see table 4) and hence the total program costs. Given levels of mf prevalence are based on diagnosis with 60 µL blood smears.

n.r.: not relevant, because costs of drugs, which are all donated, are not included in the cost projections.

a baseline assumptions.

**Table 6:** Sensitivity analysis: impact of simulation assumptions on the relative cost of semiannual/annual mass drug administration (MDA).

			Ratio of total program costs, with between brackets the number of MDA rounds required for elimination, semiannual/annual					
Region			India			West Africa		
Pre-control mf prevalence			7.7%	11.5%	15%	12.5%	20%	27.5%
Assumptions in simulations								
% of AW killed or permanently sterilized by								
DEC+ALB (India)	IVM+ALB (West Africa)	Random variation in % of AW killed						
65%	35%	No	0.90 (2/2)	0.91 (3/3)	0.92 (5/5)	0.85 (5/5)	0.86 (7/7)	0.96 (14/13)
50%	20%	No	0.91 (3/3)	0.91 (4/4)	1.07 (7/6)	0.98 (8/7)	0.89 (12/12)	n.a.
80%	50%	No	0.90 (2/2)	0.91 (3/3)	0.91 (4/4)	0.83 (3/3)	0.85 (5/5)	0.88 (10/10)
65%	35%	Yes, beta distribution with mean as specified and sd 0.30	0.90 (2/2)	0.69 (3/4)	0.92 (5/5)	0.85 (5/5)	0.98 (8/7)	0.96 (14/13)

The values in the table are the ratio of total program costs, for semiannual MDA/annual MDA. This ratio shows which approach is less expensive (with values <1 indicating that semiannual MDA is less expensive and vice versa), and it provides an indication of the relative differences in cost. The ratio is based on the estimated cost per round (under our baseline assumptions, table 3) and the required number of treatment rounds, which are shown between brackets in this table (for semiannual/annual MDA). Results are shown for Indian and West African settings, with varying pre-control mf prevalence levels. Coverage of MDA was assumed to be 70% per round (percentage of total population). Given levels of mf prevalence are based on diagnosis with 60 µL blood smears.

n.a. estimate not available: conditions unfavorable for elimination.

Including the costs of donated drugs changed the outcome of the cost analysis significantly. The costs per treatment round increased by a large amount (by an amount that was the same for annual and semiannual treatment), and the relative difference was reduced. While semiannual MDA remained cheaper in most Indian scenarios, it became slightly more expensive in the West African scenarios. The highest increase (17%) was seen in the West African scenario with the highest endemicity (pre-control mf prevalence of 27%), because here semiannual MDA would require one more round than annual MDA. Whether drugs are purchased for all eligibles in every round or for the percentage of people treated only (assuming that previously unused drugs were not wasted/expired), hardly affected the ratio of total program cost of semiannual over annual MDA.

Model assumptions about the percentages of adult worms killed (or permanently sterilized) by a single treatment affected the total number of treatment rounds needed to achieve elimination and therefore the estimated total program costs. However, this did not have a major impact on ratios of total program cost for semiannual vs. annual MDA programs (Table 6). Adding random variation in the percentage of adult worms killed (or permanently sterilized) sometimes led to an extra treatment round in either annual or semiannual MDA, but nevertheless semiannual MDA was still favored in this analysis.

## Discussion

Our simulations and cost calculations suggest that semiannual MDA will achieve LF elimination in about half of the time that would be required with annual MDA. Estimated total program costs were strongly driven by the required number of treatment rounds, and this in turn depended on pre-treatment endemicity levels and MDA coverage rates. However, total program costs for endemic countries (i.e. excluding the cost of donated drugs) were always lower for the semiannual MDA program or comparable.

### Cost projections

Cost calculations were based on observed data from 1996 and 2002 [9], [11], which were then adjusted to reflect current day practices and prices. The absolute cost estimates are subject to various assumptions. For the current purpose, though, the main interest is in the relative cost of semiannual vs. annual MDA, which is much less dependent on the assumptions. Key assumptions in the cost projections did not affect the conclusion that the cost of LF elimination with semiannual MDA is lower than or similar to the cost of programs with annual MDA. A high discount rate (reflecting a strong preference to delay cost to the future) favors annual MDA programs, in which the expenses are spread over a longer period and postponed further into the future. However, the efficiency gains of semiannual MDA mostly compensate for this. If the high costs of donated drugs are included in the cost estimates, the relative difference in cost per round diminishes and becomes negligible in West Africa. In West Africa, therefore, the efficiency gain no longer compensates for the effect of discounting or the need for an extra treatment round in semiannual MDA. But this situation only occurs when many MDA rounds are required because of unfavorable transmission conditions (as in our high endemic West African scenario). Slightly increased program costs may be justified in such situations, because here the positive impact of increasing MDA frequency on total program duration is very strong. We did not test the impact of future inflation with different annual inflation rates, but this would work in favor of shorter duration semiannual MDA programs, and it would tend to strengthen our conclusions.

### Model predictions

Estimates of the required duration of MDA in different settings were obtained by computer simulation, because empirical evidence from LF elimination programs is still limited. Many countries have made great strides, and some have stopped MDA, but no country that had ongoing transmission of LF in 2000 has been verified to have interrupted transmission of the infection using MDA [4]. Modeling is a powerful tool for decision making in infectious disease control [30], but predictions are subject to uncertainty [31]. An important uncertainty in our study concerns the efficacy of drugs. The sensitivity analysis showed that more treatment rounds would be required if the employed drugs are less effective than assumed and vice versa, while adding random variability in the percentage of worms killed by treatment did not influence the predicted outcomes. In any case, these assumptions equally affected predictions for semiannual and annual MDA programs and did not significantly affect the relative cost difference between the two strategies.

### Confirmation from field studies

Field studies are needed to confirm projected cost reductions that can be achieved with semiannual MDA in both regions and to assess any indirect effects that might affect the relative efficiency of annual vs. semiannual MDA. For example, the likelihood that unused medication is stored and used in subsequent rounds may be higher in semiannual than in annual MDA programs. Also, it is possible that increased treatment frequency will increase coverage rates (e.g. due to higher population awareness) and reduce systematic non-compliance (e.g. due to the fact that MDA does not always take place in the same season). Such changes could reduce the number of MDA rounds needed for elimination and further increase the efficiency of semiannual vs. annual MDA programs. But the opposite could also occur if insufficient effort is made to maintain high coverage rates.

### Generalizability

The efficiency gain in cost per treatment round achieved by shifting from annual to semiannual MDA was somewhat different for India and West Africa. This reflects differences in program organization and costing structure in the two regions [9], [11]. For example, the West African cost estimates included central administrative costs, laboratory costs, and adverse reaction monitoring, while these costs were not counted in the estimates for India. In general, the efficiency gain achieved is dependent on strategic choices (e.g. on activities to repeat and available budgets), health systems, program organization, and the local cost of different inputs. Results could be somewhat different in other settings. In the supporting information text S1, we show how the relative difference in total program costs depends on the relative difference in cost per treatment round, the required number of treatment rounds and applied discount rate.

The duration of MDA varies between regions because of differences in exposure patterns to mosquitoes, characteristics of the vector, timing of MDA, immigration of people, etc. Simulation results are therefore not directly generalizable to other areas, but this is not pertinent to the comparison of annual and semiannual MDA durations. This becomes clear when one compares results projected in this study for LF elimination programs in India and West Africa; although there are important differences between these models that result in very different estimates for the number of MDA rounds needed for elimination (generally higher in Africa), the basic conclusion that doubling MDA frequency halves the required duration of LF elimination programs and reduces total program costs is valid for both of these regions and it should also apply to other regions.

### Implications for LF elimination programs

Besides the total program costs, there are other important factors to consider in deciding whether MDA frequency should be increased. Increasing treatment frequency leads to a faster decline in the incidence of LF infection. This should increase the likelihood of achieving LF elimination by the target year of 2020, which is very relevant for countries that have not yet started their MDA programs. Incidence of clinical manifestations will also decline faster, which results in larger population health gain in terms of the total number of DALYs averted and results in increased productivity. Quantification of these extra benefits was beyond the purpose of this study. Increasing the treatment frequency and reducing program duration may also be beneficial for other reasons. E.g., shorter programs may be more politically attractive to health officials, and they would also be expected to have reduced risks of interruption due to natural disasters, political instability, or wars. Shorter programs may also reduce the risk of emergence of resistance to anthelmintics during LF elimination programs. Since albendazole and ivermectin also affect other diseases than LF, increasing the treatment frequency would increase their impact on diseases like soil-transmitted helminths and other NTD's – albeit for a shorter period.

Potential barriers for increasing the frequency of MDA are the increased cost per year and practical difficulties that may be associated with semiannual MDA. Increased annual drug requirements may exceed supplies of donated drugs. Also, more frequent MDA might overwhelm countries' capacities for delivering MDA to endemic populations, in view of already heavily burdened health systems and many competing health priorities [32]. Semiannual MDA may not be feasible in all areas due to weather, seasonal migration of populations, or logistical considerations. Other factors may play a role when LF elimination is integrated with programs for control of other neglected tropical diseases (NTDs). That is to say, how would accelerated LF elimination affect control programs for other NTDs?

Poor-performing programs, with very low treatment coverage, require relatively many treatment rounds. Increasing the treatment frequency from annually to semiannually would reduce the total program duration by about half, but not the number of treatment rounds. However, investments or strategies that increase coverage rates will improve results of annual or semiannual MDA, thereby reducing the number of treatment rounds required and the total costs (see Table 4).

### Conclusion

In summary, computer simulations suggest that the frequency of MDA – annual vs semiannual – does not strongly influence the total number of treatment rounds required to achieve LF elimination. The costs per year are higher with semiannual MDA, but total program costs (excluding donated drugs) are projected to be lower or about the same when semiannual MDA is used. The few situations where the total program costs of semiannual MDA are slightly higher are also challenging situations for LF elimination where semiannual MDA may improve the odds of success. Therefore, we expect semiannual MDA to be superior to annual MDA in most endemic settings. Considering the GPELF goal of LF elimination by 2020, semiannual MDA should be considered as a means of accelerating LF elimination in areas where it can be implemented.

## Supporting Information

Text S1 **Shifting from annual to semiannual MDA in LF elimination programs: relative efficiency gains, the number of treatment rounds required, and the discount rate determine the relative difference in total program cost.**
(PDF)
