# Supplementary material for: Modeling the Impact and Costs of Semiannual Mass Drug Administration for Accelerated Elimination of Lymphatic Filariasis
Source: PLoS Negl Trop Dis. 2013 Jan 3;7(1):e1984. doi: 10.1371/journal.pntd.0001984 (PMC3536806; doi:10.1371/journal.pntd.0001984)
Supplement: Text S1 — Shifting from annual to semiannual MDA in LF elimination programs: relative efficiency gains, the number of treatment rounds required, and the discount rate determine the relative difference in total program cost. (PDF) [file pntd.0001984.s001.pdf]

Text S1:

Shifting from annual to semiannual MDA in LF elimination programs: relative efficiency gains, the number of treatment rounds required, and the discount rate determine the relative difference in total program cost.

Stolk WA[1*], ten Bosch QA[1*+], de Vlas SJ[1], Fischer PU[2], Weil GJ[2], Goldman AS[3].

[1] Department of Public Health, Erasmus MC, University Medical Center Rotterdam, the Netherlands

[2] Infectious Diseases Division, Department of Internal Medicine, Washington University School of Medicine, St. Louis, MO, USA

[3] Department of Epidemiology and Biostatistics, The George Washington University School of Public Health and Health Services, Washington, DC, USA

[+]Current address: Department of Biological Sciences, University of Notre Dame, Notre Dame, IN, USA

* Contributed equally to the manuscript

Introduction

The relative cost of semiannual over annual MDA depend on the following factors, namely:

1. the relative efficiency gains achieved by implementing MDA semiannual, which can be quantified by the ratio of cost per treatment round for semiannual to annual MDA programs;
2. the number of treatment rounds needed in semiannual and annual MDA programs to achieve elimination;
3. the discount rate (see below for explanation).

Here we show how these factors, independent of a specific setting and absolute costs of MDA programs, determine the relative difference in total program costs between annual and semiannual MDA programs. Based on the tables presented in this document, the results of our study can be translated to other settings where efficiency gains or the number of treatment rounds required may be different.

Ad 1. Efficiency gains

The ratio of cost per treatment round for semiannual to annual MDA programs was estimated in this study for India and Burkina Faso. This ratio will vary between countries, because of different circumstances (e.g. distances to be travelled) or differences in relative costs of the various cost inputs (transportation, personnel, materials, etc.), but also because it depends on policy choices (which activities will be repeated, besides the drug distribution itself, and how much budget is made available). The tables in this supplement show how this ratio of cost per treatment influences the relative difference in total program cost between semiannual and annual MDA programs. This is done for a wide range of values: from the one extreme case where the second round of treatment is provided without any extra cost (average cost per round are reduced by 50% if treatment is given semiannual instead of annually), to the other extreme were all costs are doubled (the average cost per round are equal in semiannual and annual MDA programs).

Ad 3. Discounting, discount rate: brief explanation

The following text is modified from the description provided in self study materials on the website of the U.S. National Library of Medicine, National Institutes of Health (see http://www.nlm.nih.gov/nichsr/edu/healthecon/glossary.html)

Discounting is used to adjust the results for time preference in the expenditures: individuals prefer to delay costs rather than incur them in the present (assuming that they will forego a part of the benefits from the money, if it is used now). Discounting is a technique to calculate the present value of costs incurred in the future, in view of this time preference: cost incurred in the future are given a lower value. The discount rate determines by how much lower the present day value, reflecting the strength of the time preference. For example, an 100 dollar expenditure somewhere in the future, may be valued at 90 dollars at present (in other words: we are indifferent in choosing whether we have to spend 90 dollars now or 100 dollars in the future).

Legend to the tables below

Ratio of total program cost for semiannual MDA / annual MDA, in relation to the number of treatment rounds required for elimination and the efficiency gain achieved in semiannual MDA. The efficiency gain achieved in semiannual MDA is expressed as the gain relative cost per treatment round for semiannual MDA, compared to annual MDA. A value < 1 means that semiannual MDA is less expensive than annual MDA. For quick interpretation, we use color shades in the tables. Green: total program costs are lower for semiannual MDA than for annual MDA (ratio <0.95); orange: total program costs are comparable for semiannual and annual MDA (0.95 =< ratio < 1.05); red: total program costs are higher for semiannual MDA than for annual MDA (ratio >1.05). Tables A-C show the results for varying discount rates (0%, 3%, 6%).

A. Discount rate = 0%

Ratio of total programme cost for semiannual MDA / annual MDA, in relation to the number of treatment rounds required for elimination and the efficiency gain achieved in semiannual MDA											
No. of treatment rounds required for elimination (annual = semiannual)	Efficiency gain (relative cost per treatment round for semiannual MDA, compared to annual MDA)										
	**0.50**	**0.55**	**0.60**	**0.65**	**0.70**	**0.75**	**0.80**	**0.85**	**0.90**	**0.95**	**1.00**
**1**	0.50	0.55	0.60	0.65	0.70	0.75	0.80	0.85	0.90	0.95	1.00
**2**	0.50	0.55	0.60	0.65	0.70	0.75	0.80	0.85	0.90	0.95	1.00
**3**	0.50	0.55	0.60	0.65	0.70	0.75	0.80	0.85	0.90	0.95	1.00
**4**	0.50	0.55	0.60	0.65	0.70	0.75	0.80	0.85	0.90	0.95	1.00
**5**	0.50	0.55	0.60	0.65	0.70	0.75	0.80	0.85	0.90	0.95	1.00
**6**	0.50	0.55	0.60	0.65	0.70	0.75	0.80	0.85	0.90	0.95	1.00
**7**	0.50	0.55	0.60	0.65	0.70	0.75	0.80	0.85	0.90	0.95	1.00
**8**	0.50	0.55	0.60	0.65	0.70	0.75	0.80	0.85	0.90	0.95	1.00
**9**	0.50	0.55	0.60	0.65	0.70	0.75	0.80	0.85	0.90	0.95	1.00
**10**	0.50	0.55	0.60	0.65	0.70	0.75	0.80	0.85	0.90	0.95	1.00
**11**	0.50	0.55	0.60	0.65	0.70	0.75	0.80	0.85	0.90	0.95	1.00
**12**	0.50	0.55	0.60	0.65	0.70	0.75	0.80	0.85	0.90	0.95	1.00
**13**	0.50	0.55	0.60	0.65	0.70	0.75	0.80	0.85	0.90	0.95	1.00
**14**	0.50	0.55	0.60	0.65	0.70	0.75	0.80	0.85	0.90	0.95	1.00
**15**	0.50	0.55	0.60	0.65	0.70	0.75	0.80	0.85	0.90	0.95	1.00
**16**	0.50	0.55	0.60	0.65	0.70	0.75	0.80	0.85	0.90	0.95	1.00
**17**	0.50	0.55	0.60	0.65	0.70	0.75	0.80	0.85	0.90	0.95	1.00
**18**	0.50	0.55	0.60	0.65	0.70	0.75	0.80	0.85	0.90	0.95	1.00
**19**	0.50	0.55	0.60	0.65	0.70	0.75	0.80	0.85	0.90	0.95	1.00
**20**	0.50	0.55	0.60	0.65	0.70	0.75	0.80	0.85	0.90	0.95	1.00

B. Discount rate 3%

Ratio of total programme cost for semiannual MDA / annual MDA, in relation to the number of treatment rounds required for elimination											
No. of treatment rounds required for elimination (annual = semiannual)	Efficiency gain (relative cost per treatment round for semiannual MDA, compared to annual MDA)										
	**0.50**	**0.55**	**0.60**	**0.65**	**0.70**	**0.75**	**0.80**	**0.85**	**0.90**	**0.95**	**1.00**
**1**	0.50	0.55	0.60	0.65	0.70	0.75	0.80	0.85	0.90	0.95	1.00
**2**	0.51	0.56	0.61	0.66	0.71	0.76	0.81	0.86	0.91	0.96	1.01
**3**	0.51	0.56	0.61	0.66	0.71	0.76	0.82	0.87	0.92	0.97	1.02
**4**	0.51	0.57	0.62	0.67	0.72	0.77	0.82	0.88	0.93	0.98	1.03
**5**	0.52	0.57	0.62	0.67	0.72	0.78	0.83	0.88	0.93	0.98	1.04
**6**	0.52	0.57	0.63	0.68	0.73	0.78	0.84	0.89	0.94	0.99	1.04
**7**	0.53	0.58	0.63	0.68	0.74	0.79	0.84	0.89	0.95	1.00	1.05
**8**	0.53	0.58	0.64	0.69	0.74	0.79	0.85	0.90	0.95	1.01	1.06
**9**	0.53	0.59	0.64	0.69	0.75	0.80	0.85	0.91	0.96	1.01	1.07
**10**	0.54	0.59	0.64	0.70	0.75	0.81	0.86	0.91	0.97	1.02	1.07
**11**	0.54	0.59	0.65	0.70	0.76	0.81	0.86	0.92	0.97	1.03	1.08
**12**	0.54	0.60	0.65	0.71	0.76	0.82	0.87	0.93	0.98	1.03	1.09
**13**	0.55	0.60	0.66	0.71	0.77	0.82	0.88	0.93	0.99	1.04	1.10
**14**	0.55	0.61	0.66	0.72	0.77	0.83	0.88	0.94	0.99	1.05	1.10
**15**	0.55	0.61	0.67	0.72	0.78	0.83	0.89	0.94	1.00	1.05	1.11
**16**	0.56	0.61	0.67	0.73	0.78	0.84	0.89	0.95	1.01	1.06	1.12
**17**	0.56	0.62	0.67	0.73	0.79	0.84	0.90	0.96	1.01	1.07	1.12
**18**	0.57	0.62	0.68	0.74	0.79	0.85	0.91	0.96	1.02	1.08	1.13
**19**	0.57	0.63	0.68	0.74	0.80	0.85	0.91	0.97	1.03	1.08	1.14
**20**	0.57	0.63	0.69	0.75	0.80	0.86	0.92	0.97	1.03	1.09	1.15

C. Discount rate 6%

Ratio of total programme cost for semiannual MDA / annual MDA, in relation to the number of treatment rounds required for elimination											
No. of treatment rounds required for elimination (annual = semiannual)	Efficiency gain (relative cost per treatment round for semiannual MDA, compared to annual MDA)										
	**0.50**	**0.55**	**0.60**	**0.65**	**0.70**	**0.75**	**0.80**	**0.85**	**0.90**	**0.95**	**1.00**
**1**	0.50	0.55	0.60	0.65	0.70	0.75	0.80	0.85	0.90	0.95	1.00
**2**	0.51	0.57	0.62	0.67	0.72	0.77	0.82	0.87	0.93	0.98	1.03
**3**	0.52	0.57	0.62	0.68	0.73	0.78	0.83	0.88	0.93	0.99	1.04
**4**	0.53	0.58	0.63	0.69	0.74	0.79	0.85	0.90	0.95	1.01	1.06
**5**	0.53	0.59	0.64	0.70	0.75	0.80	0.86	0.91	0.96	1.02	1.07
**6**	0.54	0.60	0.65	0.71	0.76	0.82	0.87	0.92	0.98	1.03	1.09
**7**	0.55	0.60	0.66	0.71	0.77	0.82	0.88	0.93	0.99	1.04	1.10
**8**	0.56	0.61	0.67	0.73	0.78	0.84	0.89	0.95	1.00	1.06	1.12
**9**	0.56	0.62	0.68	0.73	0.79	0.85	0.90	0.96	1.02	1.07	1.13
**10**	0.57	0.63	0.69	0.74	0.80	0.86	0.92	0.97	1.03	1.09	1.14
**11**	0.58	0.64	0.69	0.75	0.81	0.87	0.93	0.98	1.04	1.10	1.16
**12**	0.59	0.65	0.70	0.76	0.82	0.88	0.94	1.00	1.06	1.11	1.17
**13**	0.59	0.65	0.71	0.77	0.83	0.89	0.95	1.01	1.07	1.13	1.19
**14**	0.60	0.66	0.72	0.78	0.84	0.90	0.96	1.02	1.08	1.14	1.20
**15**	0.61	0.67	0.73	0.79	0.85	0.91	0.97	1.03	1.09	1.15	1.21
**16**	0.61	0.68	0.74	0.80	0.86	0.92	0.98	1.04	1.11	1.17	1.23
**17**	0.62	0.68	0.75	0.81	0.87	0.93	0.99	1.06	1.12	1.18	1.24
**18**	0.63	0.69	0.75	0.82	0.88	0.94	1.01	1.07	1.13	1.19	1.26
**19**	0.63	0.70	0.76	0.82	0.89	0.95	1.02	1.08	1.14	1.21	1.27
**20**	0.64	0.71	0.77	0.83	0.90	0.96	1.03	1.09	1.16	1.22	1.28
